# Timing of administration of epinephrine predicts the responsiveness to epinephrine in norepinephrine-refractory septic shock: a retrospective study

**DOI:** 10.1186/s40560-019-0377-1

**Published:** 2019-04-05

**Authors:** Daisuke Kasugai, Mitsuaki Nishikimi, Kazuki Nishida, Michiko Higashi, Takanori Yamamoto, Atsushi Numaguchi, Kunihiko Takahashi, Shigeyuki Matsui, Naoyuki Matsuda

**Affiliations:** 1 Department of Emergency and Critical Care, Nagoya, University Graduate School of Medicine, Tsurumai-cho 64, Syowa-ku, Nagoya, Aichi 4668560 Japan; 2 Department of Biostatistics, Nagoya University Graduate School of Medicine, Tsurumai-cho 64, Syowa-ku, Nagoya, Aichi 4668560 Japan

**Keywords:** Refractory septic shock, Sepsis, Norepinephrine, Epinephrine

## Abstract

**Background:**

Currently, the appropriate method of management of patients with refractory septic shock remains unclear. This study aimed to evaluate the factors associated with response to epinephrine in norepinephrine-refractory septic shock.

**Methods:**

A retrospective single-center observational study was performed using data from adult patients (≥ 18 years old) admitted to our emergency and medical intensive care unit (ICU) from January 2014 to December 2017 who had received epinephrine to treat norepinephrine-refractory septic shock. The response was considered positive if there was increase in mean arterial pressure of 10 mmHg or decrease in arterial lactate level 3 h after epinephrine administration.

**Results:**

Forty-one patients were included: 24 responders (59%) and 17 non-responders (41%). Responders showed higher rate of survival from shock (92% vs. 18%; *P* < 0.001), and 28-day survival (83% vs. 18%; *P* < 0.001). In multivariable analysis, time of epinephrine administration after ICU admission (odds ratio [OR] 0.48; 95% confidence interval [CI] 0.27–0.87; *P* = 0.011) and SOFA score (OR 0.19; 95% CI 0.04–0.88; *P* = 0.034) were associated with epinephrine response. Time of epinephrine administration was also significantly associated with survival from shock (OR 0.42; *P* = 0.005) and 28-day survival (OR 0.14; *P* = 0.006), while SOFA score did not. Using inverse probability of treatment weighing (IPTW) adjustment of propensity score, epinephrine administration later than 24 h after ICU admission was associated with poor response (OR 0.07; 95% CI 0.02–0.21; *P* < 0.001).

**Conclusions:**

Early administration of epinephrine after ICU admission (i.e., within 24 h) is associated with better hemodynamic status in patients with refractory septic shock.

**Electronic supplementary material:**

The online version of this article (10.1186/s40560-019-0377-1) contains supplementary material, which is available to authorized users.

## Background

Refractory shock is defined as resistance to the standard dose of norepinephrine administered as a first-line vasopressor [1] and is the major reason for death among patients with sepsis or septic shock [2]. Despite the high mortality rate in patients with refractory septic shock [3–5], the appropriate management remains unclear.

A couple of guidelines suggest treatment options, including the use of epinephrine, for patients with septic shock who are refractory to norepinephrine [6, 7]. Nonetheless, the extent of the effectiveness of such treatments is unclear. Epinephrine was previously shown to have some beneficial effects against refractory septic shock in rats [8]. In clinical studies, Le Tulzo et al. also showed that epinephrine improves right ventricular contractility in septic shock patients who are unresponsive to dobutamine [9], while Mahmoud et al., in a randomized control trial, showed that compared to dobutamine, epinephrine effectively improved mean arterial pressure (MAP) and cardiac index in 60 septic shock patients resistant to norepinephrine [10]. Conversely, other large randomized controlled trials did not find epinephrine to be superior to other vasopressors or inotropic agents [11, 12].

One of the most important reasons that these randomized controlled trials failed to demonstrate the effectiveness of epinephrine may be the heterogeneity of patients with sepsis, as some are not responsive to epinephrine. If epinephrine responders can be identified in the earlier phases of septic shock, epinephrine could be used more effectively to treat such patients while alternative treatments can more promptly be considered for non-responders. However, no clinical trials have focused on identifying the type of patients who would obtain a greater benefit from receiving epinephrine. The aim of this study was to determine the variables that are strongly correlated with patient response to epinephrine in septic shock that is refractory to standard doses of norepinephrine.

## Materials and methods

### Study setting and population

A retrospective single-center observational study was performed using data from adult patients (≥18 years old) who were admitted to our emergency and medical intensive care units (EMICUs) between January 2014 and December 2017. This study was approved by the Research Ethics Committee of Nagoya University, Nagoya, Japan. Nagoya University Hospital is a quaternary academic medical center with 1035 beds, including 10 EMICU and 16 surgical ICU beds.

Eligible subjects were patients with norepinephrine-refractory septic shock who had received epinephrine after admission. Sepsis was defined according to the sepsis-3 definition [13]. Septic shock was defined as a suspected infection with shock status despite volume resuscitation and vasopressor administration, as well as a serum lactate level > 2 mmol/L after ICU admission. We defined norepinephrine-refractoriness as persistent hypotension despite the administration of approximately 0.2 μg/kg/min of norepinephrine. Persistent hypotension was defined as MAP less than 65 mmHg at the time of epinephrine initiation. Patients experiencing sepsis-associated cardiac arrest and those who required mechanical circulatory support (e.g., intra-aortic balloon pumping, venous-arterial extracorporeal membrane oxygenation [VA-ECMO], or a left ventricular assisted device) prior to epinephrine administration were excluded, as were patients with do-not-resuscitate orders before ICU admission.

### Variables of interest and outcome measurement

All data were retrospectively collected from electronic medical charts, including general characteristics (age, sex, and body mass index) and clinical information such as the suspected focus of infection, hemodynamic parameters, and laboratory data. The sequential organ failure assessment (SOFA) score and acute physiology and chronic health evaluation (APACHE) II score was calculated using the worst values obtained within 24 h after admission. At the time of epinephrine initiation, the following variables were collected; heart rate, systolic blood pressure, MAP, diastolic blood pressure, pulse pressure, central venous pressure, dose of norepinephrine, arterial blood gas data (lactate and pH), and the PaO_2_-FiO_2_ ratio. The following variables were also recorded: time from ICU admission to epinephrine administration (time-epi), adjunctive treatment (corticosteroid use, continuous renal replacement therapy [CRRT], vasopressin administration, and the use of mechanical circulatory support after epinephrine administration), fluid volume administrated during the initial 3 h of epinephrine initiation, mean initial 3-h dose of epinephrine, and MAP changes 3 h after epinephrine initiation (ΔMAP). The primary endpoint was a hemodynamic improvement after epinephrine administration, as described below. The secondary endpoints were survival from shock after epinephrine administration as described below and 28-day survival.

### Epinephrine administration and co-intervention for septic shock patients

Epinephrine administration was decided by the attending physician in the EMICU according to the current Japanese guideline for the management of septic shock [6]. Briefly, patients with septic shock who were hypotensive despite fluid resuscitation and the administration of 0.2 μg/kg/min or higher dose of norepinephrine, and when other attempts failed to improve blood pressure. Vasopressin at a dose of 0.02–0.03 units/min was administered when appropriate at least 2 h prior to epinephrine initiation. Low-dose corticosteroids (200 mg/day hydrocortisone or 40 mg/day methylprednisolone) were also considered if appropriate prior to epinephrine use. When shock was medically uncontrollable and cardiac arrest was impending, VA-ECMO was considered in some cases.

### Definition of hemodynamic improvement and survival from shock after epinephrine administration

Patients were deemed epinephrine responders if they achieved a MAP increase of 10 mmHg or their lactate level decreased 3 h after epinephrine initiation; the 3-h time point was based on a prior study [14]. When there were no 3-h lactate level data available in the electronic chart, patients were evaluated only based on ΔMAP. Survival from shock was defined as 7-day survival after epinephrine initiation without mechanical circulatory support. The duration of 7 days was chosen since previous studies had reported that median time of death due to refractory shock was 3 days [2, 15].

### Statistical analysis

Continuous data are summarized as mean ± standard deviations or median and interquartile range (25th–75th percentiles), as appropriate, depending on their distribution. Categorical variables are summarized as *n* (%). Univariate comparison of continuous variables between responders and non-responders were analyzed using the Mann-Whitney *U* test, and categorical variables were assessed using Fisher’s exact test. As for the main endpoint analysis, multivariate logistic analysis was performed to identify independent predictors of hemodynamic improvement. Clinically significant variables, namely, SOFA score ≥ 14, age, serum lactate level, log-transformed value of time-epi (log time-epi), and the use of steroids, were candidate factors; those significant on univariate analysis were subjected to a multivariate logistic regression model. We determined the logarithmic transformation of time-epi to fit the linear regression model based on its distribution (Additional file 1: Figure S1). We also converted the SOFA score into categorical variables; 14 was used as the cutoff based on a previous study [3]. For secondary endpoint analysis, the association between the variables and 7-day survival was assessed using a multivariate logistic regression model. To evaluate the robustness of the time-epi findings, we performed logistic regression analysis with inverse probability of treatment weighting (IPTW) using the propensity score [16] adjusted for severity (SOFA score), age, lactate, and steroid use. Odds ratios (OR) together with their 95% confidence intervals (CIs) were estimated in these models. All statistical tests were 2-sided, and *P* < 0.05 was considered statistically significant. All statistical analyses were performed with the R software (version 3.4.3) and EZR software (version 1.36) (Saitama Medical Center, Jichi Medical University, Saitama, Japan) [17].

## Results

### Baseline characteristics

**Fig. 1:**
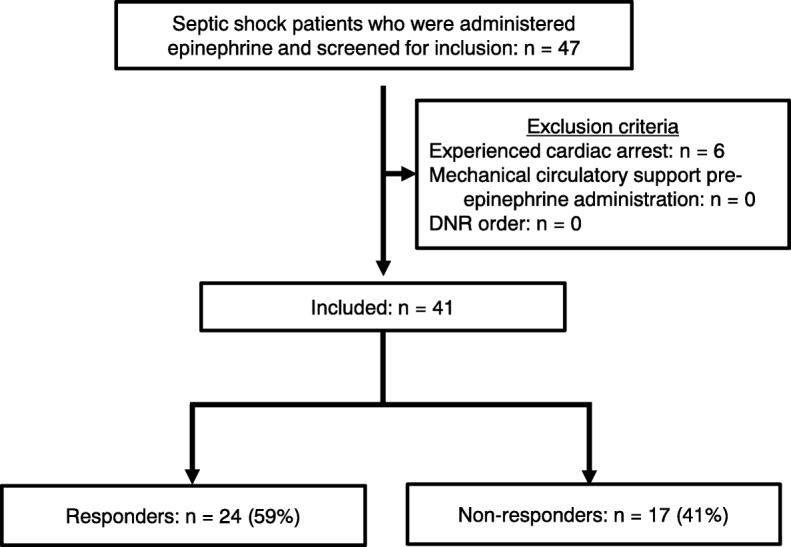
Flowchart of patient selection in this study. DNR, do-not-resuscitate

Of the 3387 patients admitted to the EMICU, 47 had experienced norepinephrine-refractory septic shock who had received epinephrine after admission. Of this number, 41 patients were included in this study, after the exclusion of 6 patients in whom epinephrine was administered after cardiac arrest. We divided the 41 patients into those who responded to the administration of epinephrine (responders; *n* = 24) and those who did not (non-responders; *n* = 17) (Fig. 1). The baseline characteristics of all 41 patients are summarized in Table 1. The average overall age of patients was 63.9 ± 15.2 years, and the median SOFA score within 24 h of ICU admission was 15 (12–16). The median norepinephrine dosage at the time of epinephrine infusion was 0.2 (0.17–0.25) μg/kg/min, and the median time-epi was 24 (12–72) h. The median fluid volume infused in the initial 3 h after epinephrine administration was 559 (426–808) ml of crystalloid. One patient in the non-responders group also received 250 ml of 5% albumin solution during the 3-h evaluation. Thirty-five patients (85%) received corticosteroid, while 20 (49%) received vasopressin, and 28 (68%) received CRRT prior to epinephrine administration. VA-ECMO by means of mechanical circulatory support for septic cardiomyopathy was introduced in 3 patients. Responders had a higher rate of survival from shock (92% vs. 18%, *P* < 0.001), and 28-day survival (83% vs. 18%, *P* < 0.001).

**Table 1:** Patient characteristics

Characteristics	Total (41)	Responders (24)	Non-responders (17)	*P* value
At the day of ICU admission				
Age, years	63.9 ± 15.2	65.3 ± 13.3	61.8 ± 17.6	0.476
Male, *n* (%)	25 (60)	15 (63)	10 (59)	> 0.999
BMI	21.8 ± 5.8	22.1 ± 7.0	21.3 ± 3.8	0.657
SOFA score	15 (12–16)	13 (12–15)	16 (14–19)	0.009
APACHE II score	33 (29–39)	36 (30–39)	32 (27–37)	0.597
Creatinine, mg/dL	1.46 (0.93–2.29)	1.82 (0.92–2.39)	1.20 (0.99–1.71)	0.327
Bilirubin, mg/dL	1.4 (0.9–4.4)	1.05 (0.8–1.6)	1.7 (1.1–7.3)	0.065
Platelet count	71 (44–119)	105 (54–176)	50 (25–71)	0.022
CRP, mg/dL	10.5 (6.2–18.5)	9.83 (5.9–15.3)	10.5 (6.4–22.9)	0.666
PCT, ng/mL	9.4 (1.4–51.9)	17.1 (1.8–52.9)	5.70 (1.2–27.2)	0.39
WBC × 10^3^/μL	10.7 (3.7–17.1)	14.0 (5.1–19.5)	7.4 (1.2–13.1)	0.153
Hct	29.8 (28.2–32.2)	30.8 (29.1–32.5)	28.6 (27.5–29.9)	0.09
PT-INR	1.38 (1.16–1.7)	1.39 (1.15–1.74)	1.29 (1.23–1.58)	0.624
FDP, μg/ml	16.4 (11.1–38.3)	14.5 (9.8–32.1)	22.6 (3.0–50.6)	0.321
BNP, pg/ml	432 (119–1168)	463 (128–1169)	248 (119–961)	0.767
AT3 activity, %	54 (45–66)	49 (39–63)	59 (50–75)	0.068
Focus of infection				
Soft tissue, *n*	5	5	0	0.065
Respiratory, *n*	10	7	3	0.48
Central nervous system, *n*	2	1	1	> 0.999
Gastrointestinal, *n*	3	1	2	> 0.999
Hepatobiliary, *n*	5	2	3	0.633
Bacteremia or other, *n*	16	9	7	> 0.999
At the time of epinephrine infusion				
MAP, mmHg	51.3 ± 11.2	52.1 ± 10	50.3 ± 13	0.613
PP, mmHg	24.7 ± 12	26.3 ± 11	22.5 ± 13	0.317
HR, bpm	107 ± 21	103 ± 21	113 ± 20	0.149
CVP, mm Hg	15 (12–17)	13 (11–17)	15 (13–18)	0.2
Lactate, mmol/L	4.4 (2.6–8.9)	3.4 (2.1–7.6)	6.0 (3.6–9.6)	0.098
pH	7.309 (7.386–7.181)	7.336 (7.212–7.386)	7.261 (7.098–7.386)	0.266
P-F ratio	163 (92–302)	222 (111–354)	131 (83–164)	0.093
Norepinephrine, μg/kg/min	0.2 (0.17–0.25)	0.19 (0.17–0.23)	0.25 (0.16–0.28)	0.467
Epinephrine, μg/kg/min*	0.07 (0.03–0.1)	0.05 (0.03–0.1)	0.08 (0.07–0.14)	0.068
Time-epi, h**	24 (12–72)	18 (12–24)	72 (6.0–144)	0.004
Adjunctive therapy				
Fluid infusion, ml***	559 (426–808)	559 (418–762)	559 (447–958)	0.989
Corticosteroid, *n* (%)	35 (85)	20 (83)	15 (88)	> 0.999
Vasopressin, i (%)	20 (49)	10 (42)	10 (59)	0.35
CRRT, *n* (%)	28 (68)	15 (63)	13 (76)	0.505
VA-ECMO, *n* (%)	3 (7)	0 (0)	3 (18)	0.006
Endpoints				
ΔMAP after initiation, mmHg ****	13 (0–22)	20 (14–29)	-1 (− 7 to 6)	< 0.001
Lactate 3 h after initiation, mmol/L	6.4 (3.1–11.5)	4.2 (2.8–9.0)	11.4 (5.1–13.8)	0.039
Survival from shock, *n* (%) *****	25 (61)	22 (92)	3 (18)	< 0.001
28-day survival, *n* (%)	23 (56)	20 (83)	3 (18)	< 0.001

*ICU* intensive care unit, *BMI* body mass index, *SOFA* Sequential Organ Failure Assessment, *APACHE* acute physiology and chronic health evaluation, *CRP* C-reactive protein, *PCT* procalcitonin, *WBC* white blood cell, *PT-INR*: prothrombin time-international normalized ratio, *FDP*: fibrin degradation product, *BNP* brain natriuretic peptide, *AT3* anti-thrombin 3, *MAP* mean arterial pressure, *PP* pulse pressure, *HR* heart rate, *CVP* central venous pressure, *CRRT* continuous renal replacement therapy, *VA-ECMO* veno-arterial membrane oxygenation

*Median epinephrine dose of initial 3-h administration

**Time of epinephrine administration after ICU admission

***Fluid administration during initial 3 h of epinephrine use

****MAP change 3 h after epinephrine initiation

*****7-day survival without the use of mechanical circulatory support

Data are presented as the mean ± standard deviation, the median and interquartile ranges (25–75% percentile), or as absolute frequencies with percentages

### Factors associated with responsiveness to epinephrine administration

We performed univariate analysis to identify factors that were strongly associated with responsiveness to the administration of epinephrine (Table 2). The SOFA score within 24 h of ICU admission and the log time-epi were significantly associated with responsiveness to epinephrine. We subjected these 2 variables to multivariate logistic regression analysis, which showed them to be independently associated with responsiveness to epinephrine (OR 0.48, 95% CI 0.27–0.87, *P* = 0.011 and OR 0.19, 95% CI 0.04–0.88, *P* = 0.034, respectively) (Table 3). To evaluate the robustness of time-epi as a factor, we also investigated the OR of epinephrine response upon its later administration using the IPTW-propensity score, which was 0.07 (95% CI 0.02–0.21; *P* = 0.001) (Table 3). This suggested that epinephrine administration within 24 h of ICU admission was significantly associated with a better response. Furthermore, we found that the log time-epi was significantly associated with survival from shock and 28-day survival, whereas the SOFA score was not (Table 4).

**Table 2:** Univariate analyses of factors predicting epinephrine response

Variables	OR	95% CI	*P* value
SOFA score ≥ 14	0.21	0.05–0.8	0.022
Age	1.02	0.97–1.06	0.467
Lactate	0.91	0.8–1.05	0.19
Log time-epi	0.21	0.06–0.73	0.013
Steroid	0.67	0.11–4.13	0.663

*OR* odds ratio, *CI* confidence interval, *SOFA* Sequential Organ Failure Assessment, *time-epi* time of epinephrine administration after intensive care unit admission

**Table 3:** Multivariable logistic regression analyses of predicting factors of epinephrine response

Variable	OR (95% CI)	*P* value
Log time-epi	0.48 (0.27–0.84)	0.011
SOFA score ≥ 14	0.19 (0.04–0.88)	0.034
Adjusted using IPTW-propensity score*		
Time-epi ≥ 24 h	0.07 (0.02–0.21)	< 0.001

*OR* odds ratio, *CI* confidence interval, *Time-epi* time of epinephrine administration after intensive care unit admission, *SOFA* Sequential Organ Failure Assessment, *IPTW* inverse probability of treatment weighting

*Variables used to calculate the propensity score included age, lactate, steroid use, and SOFA score

**Table 4:** Multivariable logistic analyses of predicting factors for survival from shock and 28-day survival

Variable	OR (95% CI)	*P* value
Association with survival from shock*		
Log time-epi	0.42 (0.23–0.77)	0.005
SOFA score ≥ 14	0.41 (0.09–1.84)	0.247
Association with 28-day survival		
Log time-epi	0.14 (0.04–0.57)	0.006
SOFA score ≥ 14	0.6 (0.14–2.53)	0.488

*OR* odds ratio, *CI* confidence interval, *Time-epi* time of epinephrine administration after intensive care unit admission, *SOFA* Sequential Organ Failure Assessment

*7-day survival without the use of mechanical circulatory support

## Discussion

A major reason for the efficacy of epinephrine in treating refractory septic shock not being clearly established is that previous studies included the entire septic shock patient population at a given facility, despite the fact that some patients respond more favorably to epinephrine than others. In the present study, we retrospectively analyzed the data of 41 septic patients who were refractory to norepinephrine and found that the timing of epinephrine administration, as well as the SOFA score within the first 24 h of ICU admission, was strongly associated with responsiveness to epinephrine.

### Relationship between the response to epinephrine, the timing of its administration, and the SOFA score

Our finding that late-stage epinephrine administration was associated with low response rate may be explained by the fact that catecholamine resistance in patients with sepsis changes over time. This change is caused by intracellular calcium overload and impairment of intracellular signaling via adrenergic beta receptors in cardiomyocytes [18, 19]. If epinephrine is administered in the later stages of septic shock, the increased catecholamine resistance may reach a level that prevents any response to epinephrine. To the best of our knowledge, ours is the first study to show that the time course of septic shock is associated with the hemodynamic response to catecholamines. Furthermore, our study confirmed a previous finding by Conrad et al. that a higher SOFA score is strongly associated with catecholamine hypo-responsiveness [3].

Interestingly, IPTW analysis revealed that the administration of epinephrine within 24 h improved responsiveness compared to later administration. A randomized control trial failed to show any efficacy of epinephrine administration in treating septic shock, which might be because in that study the mean time for patient randomization after ICU admission was 48 h [12], which appears to be too long to observe a benefit for epinephrine administration and more likely to observe a lack of response to epinephrine administration. Our findings may provide insight into the reason negative data were produced in the abovementioned study.

### The analysis of secondary outcome

While 28-day survival is commonly used in the field of critical care, we also chose 7-day survival after epinephrine initiation without mechanical circulatory support as the secondary outcome to focus on cause-specific mortality of refractory shock and to make sure of consistency in the findings between primary and secondary endpoint analyses. The reason for death in sepsis is multifactorial [2]. Several studies have reported that the median time to death in refractory shock was 3 days [2, 15]. Therefore, we consider that 7 days were long enough to detect death due to shock. VA-ECMO might be an effective rescue therapy for refractory shock in some septic shock patients [20]. In our study group, 3 patients who received VA-ECMO therapy were considered hemodynamically unstable after using epinephrine. We decided that they would die of shock death if ECMO was not used. Therefore, we classified those who received ECMO as non-survivors even if they survived longer than 7 days. By using this outcome, we showed that the timing of epinephrine administration was linked to some extent with the short-term efficacy of epinephrine in preventing death from septic shock. Also, we found the results of analyses were similar between secondary endpoints.

While late administration of epinephrine (> 24 h) was associated with worse short-term outcome, initial SOFA score was not. We need a larger sample size to determine whether initial SOFA score is useful in predicting the short-term patient outcome after epinephrine use. In any case, late stages of septic shock rarely benefit from epinephrine administration and should be excluded from future clinical trials.

### Effects of co-interventions over the results

One of the major limitations of this study was that confounding bias of co-interventions (fluid, corticosteroids, vasopressin) cannot be excluded, owing to the limited sample size and retrospective nature of the study. Although we used epinephrine when prior co-interventions failed, these interventions can be possible confounders. To minimize the effect of confounders, a definitive treatment protocol must be devised. It would be interesting to evaluate other indicators in epinephrine responders in future prospective studies with a certain protocol.

### Other limitations

Our study has several other limitations. First, it was a single-center retrospective observational study, and the administration of epinephrine was decided based on the clinical preferences of attending physicians. Although selection bias cannot be completely ruled out, the baseline hemodynamic parameters (MAP, heart rate, serum lactate levels, etc.) were similar between responders and non-responders. Also, we selected arbitrary endpoints for the evaluation of variables. The clinical importance of these endpoints should be validated in the future. Second, our study involved a small-sized cohort; this may explain our inability to detect a significant association between the SOFA score and short-term outcome, in contrast to a previous report [3]. Future study with a large sample size should be performed to see the effect of variables (e.g., lactate, and other laboratory data) for the hemodynamic response. Third, we did not evaluate the relationship between the site of infection and epinephrine response; this should be evaluated in a future trial. Finally, long-term outcomes were not investigated because of the study design and sample size; it would be of great interest to determine the association between epinephrine response and such long-term outcomes.

## Conclusions

Early administration of epinephrine after ICU admission (i.e., within 24 h) is associated with hemodynamic improvement in norepinephrine-refractory septic shock.

## Additional file

Additional file 1: **Figure S1.** The time distribution of epinephrine initiation after intensive care unit admission. The minimum and maximum times were 3 and 696 h, respectively, while the median (interquartile range) was 24 (12–72) h. (PPTX 1525 kb)

## Abbreviations

APACHE: Acute physiology and chronic health evaluation
CI: Confidence interval
CRRT: Continuous renal replacement therapy
EMICU: Emergency and medical intensive care unit
ICU: Intensive care unit
IPTW: Inverse probability of treatment weighting
MAP: Mean arterial pressure
OR: Odds ratio
SOFA: Sequential Organ Failure Assessment
time-epi: Time from ICU admission to epinephrine administration
VA-ECMO: Venous-arterial extracorporeal membrane oxygenation
