# Supplementary figures and images for: Timing of administration of epinephrine predicts the responsiveness to epinephrine in norepinephrine-refractory septic shock: a retrospective study
Source: J Intensive Care. 2019 Apr 5;7:20. doi: 10.1186/s40560-019-0377-1 (PMC6451296; doi:10.1186/s40560-019-0377-1)

## Slide 1
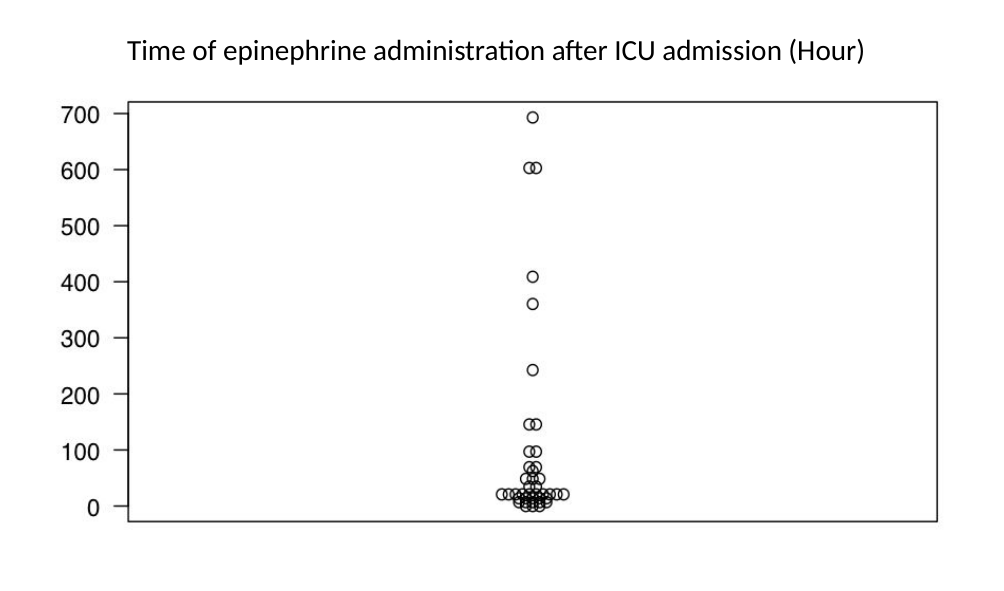

Time of epinephrine administration after ICU admission (Hour)

Supplement: Supplementary file 1 — Figure S1. The time distribution of epinephrine initiation after intensive care unit admission. The minimum and maximum times were 3 and 696 h, respectively, while the median (interquartile range) was 24 (12–72) h. (PPTX 1525 kb) [file 40560_2019_377_MOESM1_ESM.pptx]
